# 

**Published:** 1876-11

**Authors:** 


### CONTENTS:

ARTICLE I.—Anatomical and Physiological Differences between the White and Negro Races. By W. J. Burt, M. D. .......... 289

ARTICLE II.—Cases of Sarcomatous Tumor. By J. Ewing Mears, M. D. .......... 297

ARTICLE III—Crystal Gold. By Ambler Tees, D. D. S. .......... 304

ARTICLE IV.—American Academy of Dental Surgery .......... 310

ARTICLE V.—American Academy of Dental Science .......... 312

ARTICLE VI.—Boracic Acid and its Application. By Leonard Cane, M. D. .......... 316

ARTICLE VII.—Tumors and Abscesses—Causes and Treatment. By W. H. Atkinson, D. D. S. .......... 318

ARTICLE VIII.—Nervous Exhaustion .......... 323

ARTICLE IX.—Removal of Steatomatous Tumor .......... 327

Virginia State Dental Association .......... 328

### EDITORIAL, ETC.

The Dental Exhibit at the Centennial .......... 329

### MONTHLY SUMMARY.

Death from Boxing of the Ears .......... 333

Cause of Decay of the Teeth .......... 334

Anæsthesia of Drunkenness Recommended .......... 334

Female Doctors in Europe .......... 335

Death from Chloroform .......... 335

The Chemistry of Salicylic Acid .......... 336

Means to make Leeches take Immediately .......... 336

